# Activity of *N*-Chlorotaurine against Periodontal Pathogens

**DOI:** 10.3390/ijms25158357

**Published:** 2024-07-30

**Authors:** Kacper Kowalczyk, Débora C. Coraça-Huber, Walter Wille-Kollmar, Michael Berktold, Markus Nagl

**Affiliations:** 1 Institute of Hygiene and Medical Microbiology, Medical University of Innsbruck, A-6020 Innsbruck, Austria; kacperkowalczykkacper@gmail.com (K.K.); michael.berktold@i-med.ac.at (M.B.); 2 Research Laboratory for Biofilms and Implant Associated Infections (BIOFILM LAB), University Hospital for Orthopaedics and Traumatology, Medical University of Innsbruck, A-6020 Innsbruck, Austria; debora.coraca-huber@i-med.ac.at; 3 Independent Researcher, Dentist’s Practice, A-6020 Innsbruck, Austria; willekollmar@gmail.com

**Keywords:** *N*-chlorotaurine, periodontitis, peri-implantitis, active halogen compound, chloramine, antiseptic, biofilm

## Abstract

Dental plaque bacteria play an important role in the pathogenicity of periodontitis and peri-implantitis. Therefore, antimicrobial agents are one means of treatment. *N*-chlorotaurine (NCT) as an endogenous well-tolerated topical antiseptic could be of advantage for this purpose. Accordingly, its microbicidal activity against some dental plaque bacteria was investigated at therapeutic concentrations in vitro. In quantitative killing assays, the activity of NCT against planktonic bacteria and against biofilms grown for 48 h on implantation screws was tested. Electron microscopy was used to demonstrate the formation of biofilm and its morphological changes. The killing of planktonic bacteria of all tested species, namely *Streptococcus sanguinis*, *Streptococcus salivarius*, *Streptococcus oralis*, *Streptococcus cristatus*, *Rothia aeria*, and *Capnocytophaga ochracea*, was shown within 10–20 min by 1% NCT in 0.01 M phosphate-buffered saline at 37 °C. Bacteria grown on screws for 24 h were inactivated by 1% NCT after 15–20 min as well, but the formation of biofilm on the screws was visible in electron microscopy not before 48 h. The killing of biofilms by 1% NCT was demonstrated after 30 min (streptococci) and 40 min (*R. aeria*). As expected, NCT has broad activity against dental plaque bacteria as well and should be further investigated on its clinical efficacy in periodontitis and peri-implantitis.

## 1. Introduction

Oral pathogens play a pivotal role in dental plaque formation and frequent diseases in odontology such as gingivitis, periodontitis, peri-implant mucositis, and peri-implantitis. Several complexes of subgingival bacterial species have been identified, which are related to disease [1]. They may induce inflammation directly via numerous virulence factors (enzymes and toxins) or indirectly via activation of the defence system [2]. The formation of biofilm is also important for dental deterioration. Gingival cells react with the release of proinflammatory cytokines such as interleukins 1,6, and 8 and tumor necrosis factor alpha, which attracts leukocytes. An imbalance between bacteria and their virulence factors and host immunity is thought to be responsible for the persistence of inflammation and periodontal destruction [2]. Of note, the oral microbiome and periodontitis appears not only to be a local matter but may influence systemic diseases as well [2,3]. For instance, correlations between periodontitis and autoimmune diseases such as rheumatoid arthritis and collagenoses and malignancies such as colorectal carcinoma, preterm birth, diabetes, cardiovascular disease, and neurodegenerative disorders, such as Alzheimer’s disease, have been found [2,3].

Due to the role of periodontal pathogens, antimicrobial mouthwashes are in use for the treatment of periodontal disease [4]. Representatives are mainly antiseptic compounds, such as chlorhexidine, hydrogen peroxide, iodine, quaternary ammonium compounds, and essential oils in alcoholic solution [4,5]. They should be used as adjunctive therapy to oral hygiene and professional plaque removal to be effective in the reduction of periodontal pocket depth, dental plaque, and bleeding [4,6]. Transient application is suggested to avoid adverse effects [4,6].

Previously, a common concept was to obtain a microbicidal effect as strong as possible by antiseptics. More recently, however, this concept has been questioned in light of the oral microbiome, which is important not only for oral health and which should not be damaged by therapy as far as possible [3,4]. For instance, an influence of chlorhexidine on the oral microbiome with a shift to Firmicutes and Proteobacteria was shown, accompanied by a decrease in nitrite concentration and a trend of elevated blood pressure in healthy individuals [7].

Searching for antimicrobial agents which should circumvent the problem of resistance and the possible systemic effects of antibiotics and the problem of local toxicity and damage of the oral microbiome, the endogenous anti-infective and mild antiseptic *N*-chlorotaurine (NCT) might be of interest [8,9,10,11]. It is formed by the reaction of hypochlorous acid (HOCl) with taurine in activated granulocytes and monocytes during inflammation and thought to contribute to the inactivation of invading pathogens and to the termination of inflammation [12,13]. As an active chlorine compound, it has broad-spectrum activity against all kinds of pathogens (bacteria, viruses, fungi, protozoa) without the development of resistance [9,14,15]. The first results showing the activity of NCT besides N-bromotaurine and HOCl in low concentration against the periodontal pathogens *Streptococcus mutans* and *Porphyromonas gingivalis* came from the research group of Marcinkiewicz [16]. Additionally, NCT has anti-inflammatory properties such as the downregulation of proinflammatory cytokines and chemokines and the upregulation of hemoxygenase-1 [17,18,19,20] and distinct anti-inflammatory effects on leukocytes [21,22]. The possible implications of the immunological effects of HOCl and NCT in periodontal diseases were already discussed in 2004 by Mainnemare et al. [23]. Preclinical and clinical investigations on NCT were facilitated by its synthesis as a crystalline sodium salt [9]. Clinical trials demonstrated its tolerability and efficacy in the treatment of infections of the human eye, the skin, the outer ear, and oral cavity [9,24,25]. This was confirmed by phase 1 and phase 2a studies and case applications in further body regions, for instance, the upper and lower airways and the urinary bladder [9,19,26].

The effect of 2% and 3% NCT mouth rinses on plaque regrowth and vitality was investigated in an investigator-blind, randomized, and controlled study versus 0.2% chlorhexidine and 0.9% sodium chloride in human volunteers [24]. The participants rinsed with 10 mL of the allocated solution for 1 min twice daily over a total period of 4 days. NCT did not inhibit plaque regrowth in contrast to chlorhexidine but significantly reduced the vitality of plaque compared to both control groups [24]. Adverse effects consisted of a chlorine taste for a few minutes and a brownish tongue discoloration for a few days. The latter was never recorded in single cases using the standard concentration of 1% NCT as throat spray or gargling solution for the treatment of common cold [19].

In another study, pig teeth pre-treated with four different drill bits in vitro were artificially contaminated with *Staphylococcus aureus* [27]. Four irrigations of the teeth for five minutes with 1% NCT solution significantly reduced the bacterial viable count compared to 0.9% sodium chloride by ≥2 log_10_.

According to the previous work, NCT could be suited for the prophylaxis and treatment of periodontitis and peri-implantitis. Therefore, we investigated its microbicidal activity against some dental plaque bacteria in planktonic form and in biofilm.

## 2. Results

### 2.1. Activity of N-Chlorotaurine (NCT) against Planktonic Dental Plaque Bacteria in PBS Solution

**Figure 1:**
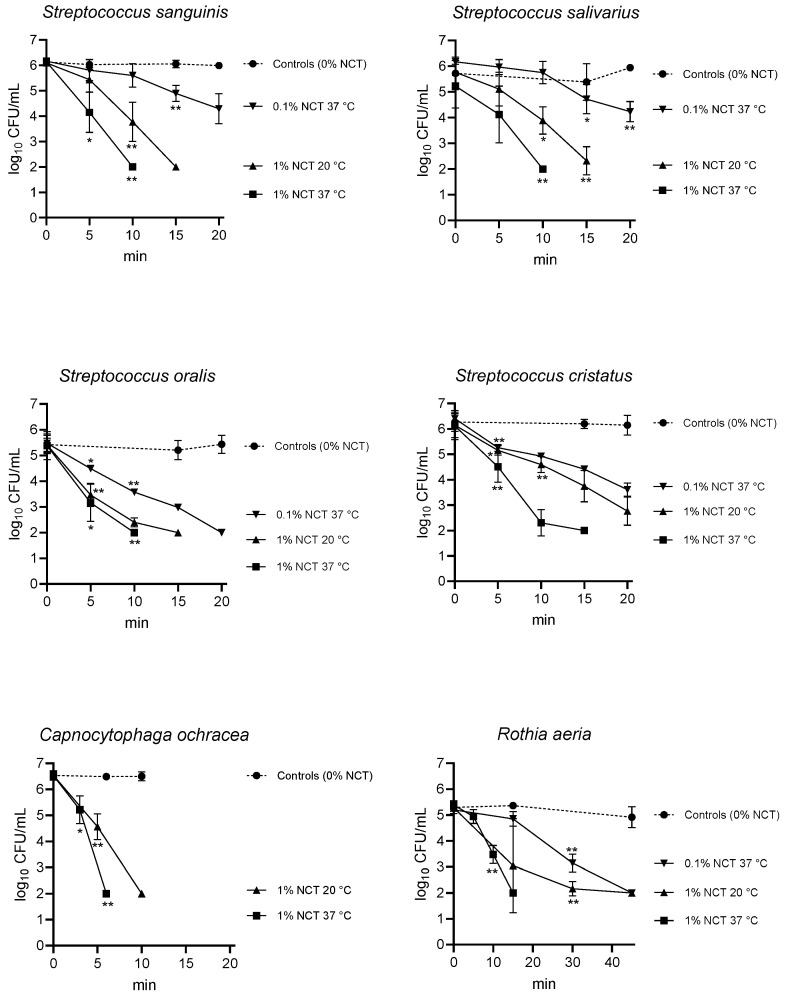
Killing of planktonic dental plaque bacteria in PBS solution at pH 7.1 by 1% NCT at 37 °C (-■-), 1% NCT at 20 °C (-▲-), and 0.1% NCT at 37 °C (-▼-). Controls in PBS without NCT (-●-, dotted lines). Mean values and SD of three independent experiments (n = 4 for *S. cristatus* 1% NCT 20 °C). Threshold values of significance versus controls are indicated. *R. aeria* needed longer incubation times for significant reduction by 0.1% NCT, which is indicated. * *p* < 0.05, ** *p* < 0.01. The detection limit was 2 log_10_ CFU/mL.

There was a clear bactericidal activity of NCT against all test bacteria, *R. aeria*, *C. ochracea*, *S. oralis*, *S. sanguinis*, *S. salivarius*, and *S. cristatus*. As shown in Figure 1, the killing curves were dependent on the concentration of NCT and on the temperature. At 37 °C, 1% (55 mM) NCT reduced the CFU count to the detection limit within 10 min with all test strains; only *R. aeria* needed 15 min for the same effect. At 20 °C, the killing by 1% was slightly slower as expected. Reduction in the concentration to 0.1% NCT had a similar effect but still caused marked inactivation of bacteria within 20–30 min. A direct comparison of all bactericidal activities of NCT derived from the whole killing curves (BA values, log_10_ reduction in CFU per min) is shown in Table 1.

### 2.2. Activity of NCT against Planktonic Dental Plaque Bacteria on Dental Implant Screws

**Figure 2:**
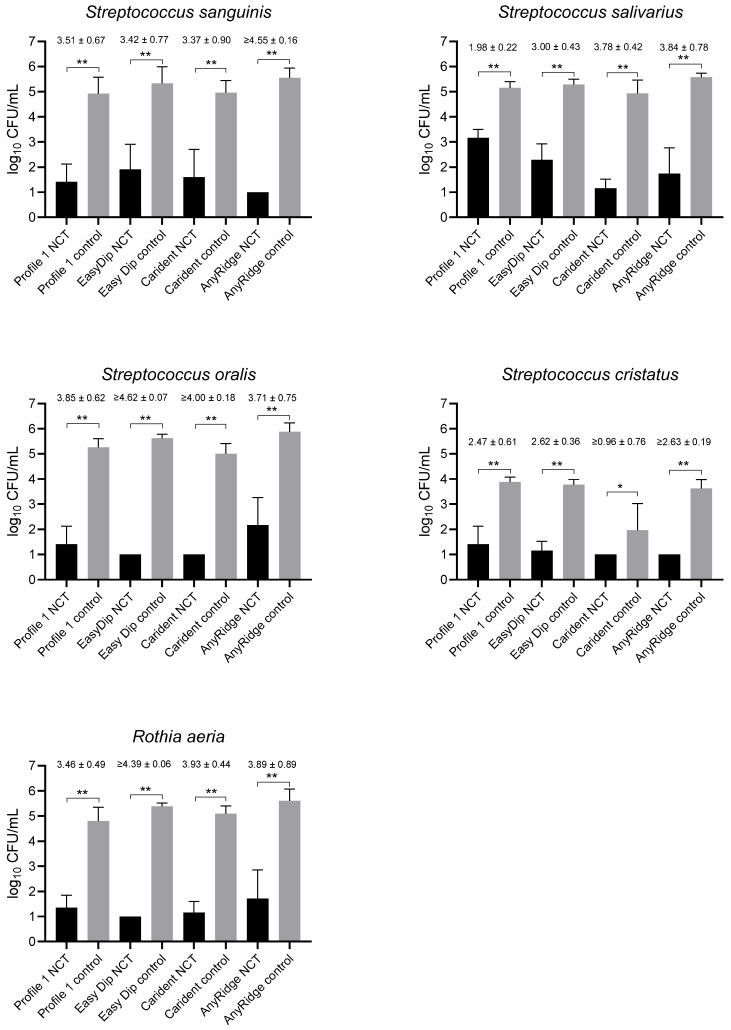
Killing of dental plaque bacteria attached to implant screws subsequent to growth in the presence of the screws for 24 h by 1% NCT incubated for 15 min (streptococci) or 20 min (*R. aeria*) at 37 °C and pH 7.1. Controls in PBS without NCT. Values of log_10_ reduction in CFU/mL are indicated for each implant material. Mean values and SD of 3–8 independent experiments (n = 3 for Profile 1, 3–6 for Easy Dip, 5–8 for Carident, 3–5 for AnyRidge). * *p* < 0.05, ** *p* < 0.01. The detection limit was 1 log_10_ CFU/mL.

Incubation of the screws, which were from four different companies as listed in Table 2, in the bacterial suspension for 24 h yielded only some small scattered areas of streptococci with a visible biofilm as evaluated by electron microscopy (see below). With *R. aeria*, no biofilm was found. Therefore, this series of experiments was regarded more as the activity of NCT against bacteria attached to screws than against biofilm. An incubation time of 15 min proved as sufficient for the standard concentration of 1% NCT at 37 °C and was chosen for all test streptococci, while it was 20 min for *R. aeria*. *C. ochracea* did not grow well in this setting over 24 h so it was not evaluated in these tests. NCT demonstrated a highly significant bactericidal activity against all tested strains cultured in the presence of the implant screws and attached to them (Figure 2). The log_10_ reduction in CFU compared to the controls reached between 3.00 and 4.55 for *S. sanguinis*, *S. salivarius*, and *S. oralis* with one exception of 1.98 for Profile 1 and *S. salivarius*. Due to significantly lower CFU counts in the controls, the log_10_ reduction came only to 0.96 to 2.63 in *S. cristatus*, although the detection limit was largely reached in the NCT samples similar to the other strains (Figure 2). High reduction values could be achieved with *R. aeria* after 20 min incubation in 1% NCT (Figure 2).

### 2.3. Activity of NCT against Biofilm of Dental Plaque Bacteria on Dental Implant Screws

**Figure 3:**
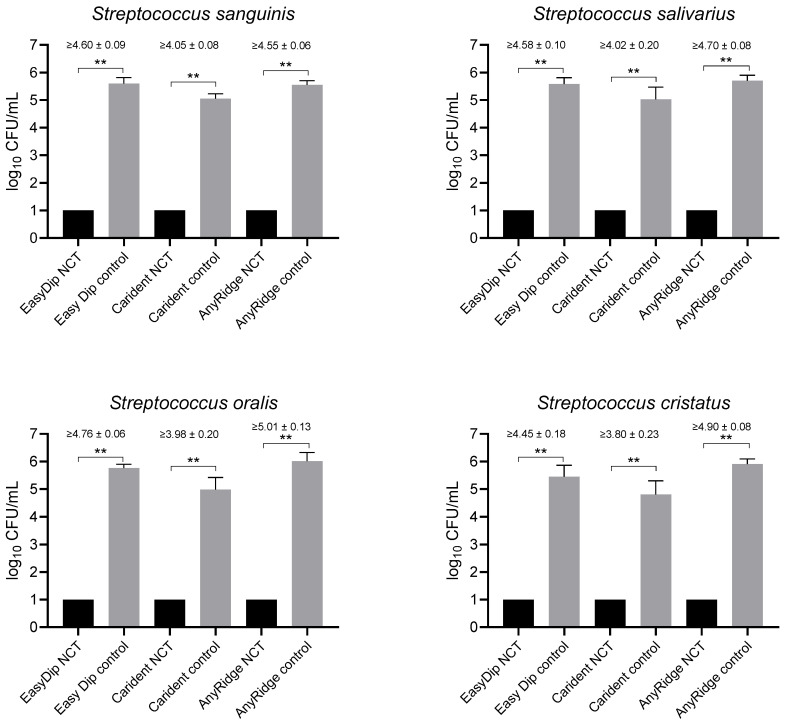
Killing of biofilms of dental plaque bacteria on implant screws subsequent to growth in the presence of the screws for 48 h by 1% NCT incubated for 30 min at 37 °C and pH 7.1. Controls in PBS without NCT. Values of log_10_ reduction in CFU/mL are indicated for each implant material. Mean values and SD of three independent experiments. ** *p* < 0.01. The detection limit was 1 log_10_ CFU/mL.

An incubation of the screws in the bacterial suspension for 48 h yielded biofilms on the screws with streptococci (see below electron microscopy). After removing the planktonic bacteria by three washing steps in PBS, 1% NCT at 37 °C killed the streptococcal biofilms to the detection limit after 30 min incubation. The log_10_ reduction in CFU/mL ranged between ≥3.80 and ≥5.01 (Figure 3). In three experiments with *R. aeria* and *C. ochracea* also, NCT led to complete killing. The controls of *R. aeria, however,* grew to 3.09 to 4.95 log_10_ CFU/mL only in 1–2 experiments in the presence of different screws with zero growth in the other experiments. With *C. ochracea*, only in one experiment, CFU/mL counts of up to 3.78 log_10_ were found in the controls. Therefore, no reliable and significant results could be obtained with these two strains.

### 2.4. Comparison of Attachment of Viable Bacteria on Different Screws

**Figure 4:**
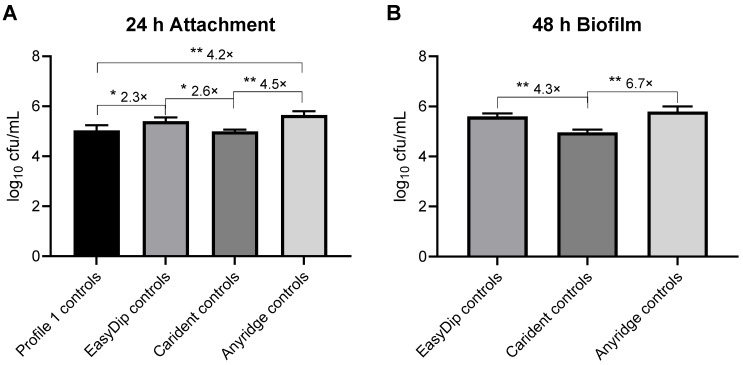
Comparison of the bacterial load on different implant screws. CFU counts of control screws incubated for 24 h (**A**) or 48 h (**B**) at 37 °C in the presence of bacteria in tryptic soy broth followed by 15 min (**A**) or 30 min (**B**) incubation in PBS. All bacterial species except for *S. cristatus* are summarized in (**A**) and all except for *R. aeria* and *C. ochracea* in (**B**). Values of log_10_ reduction in CFU/mL are indicated for each implant material. Mean values and SD of four summarized values each. * *p* < 0.05, ** *p* < 0.01 versus Carident and Profile 1 by one-way ANOVA and Tukey’s multiple comparison test and numeric values of different CFU counts are shown.

In tests with implant screws incubated for 24 h and 48 h in bacteria suspensions (Figure 2 and Figure 3), there was the impression of slightly lower bacterial count on the Carident and Profile 1 screws than on EasyDip and Anyridge ones in the controls not treated with NCT. To check for a possible difference, we calculated the average CFU counts of all bacterial species of the four independent experiments and compared these values (Figure 4). *S. cristatus* from the 24 h test and *R. aeria* and *C. ochracea* from the 48 h test were excluded because of their low counts that would have been unreliable for this comparison. As can be seen in Figure 4, Carident and Profile 1 showed up to 6.7-fold (0.828 log_10_) and 4.2-fold (0.619 log_10_), respectively, which are lower counts than EasyDip and Anyridge screws.

### 2.5. Scanning Electron Microscopy of Biofilm on Implant Screws

**Figure 5:**
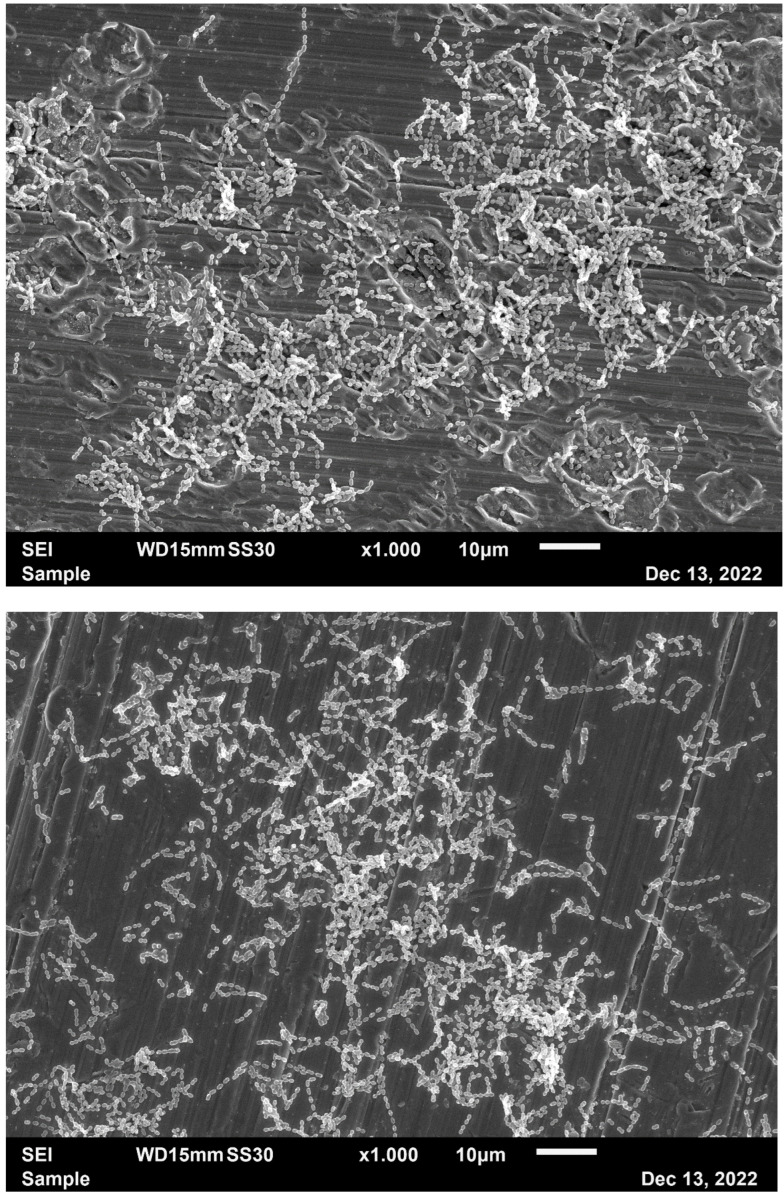
Scanning electron microscopy of a biofilm of *S. sanguinis* on implant screws after 48 h incubation in tryptic soy broth at 37 °C. The exposures were taken after subsequent incubation in PBS (**upper panel**) or 1% NCT (**lower panel**) for 30 min at 37 °C; magnification, ×1000.

With streptococci, scattered areas of biofilm could be produced on the implant screws after 24 h incubation time, while larger areas of more compact biofilm became visible after 48 h incubation in the presence of bacteria in nutrient broth. In Figure 5, a typical 48 h biofilm of *S. sanguinis* is shown after incubation in NCT or PBS control for 30 min. No clear visible difference could be detected between test and control bacteria by electron microscopy, but there was the impression of less extracellular matrix in NCT samples (Figure 5). With *R. aeria*, no biofilm could be detected after 24 h and only very few spots of biofilm after 48 h so that no reliable evaluation was possible with this pathogen.

There was less extracellular matrix in the NCT-treated biofilm.

## 3. Discussion

As a representative of active chlorine compounds, NCT has broad-spectrum microbicidal activity against microorganisms [9,14]. This has been confirmed in this study against dental plaque bacteria in planktonic and biofilm form. They were chosen since streptococci are regarded as early colonizers involved in the formation of dental plaque and building the basis for later destruction by anaerobic bacteria [1,3]. *Capnocytophaga spp*. and *Rothia spp*. are also a component of oral biofilms and associated with local and in part systemic diseases [3,28]. The activity against planktonic forms was in the range of that against other Gram-positive and Gram-negative bacteria of importance in medicine, such as staphylococci, *Streptococcus pyogenes*, Enterobacteriaceae, *Pseudomonas aeruginosa*, and others [9,15]. In pig teeth perforated with drill bits and artificially contaminated with *S. aureus*, irrigations with NCT were successful in inactivating these bacteria in an earlier study, basically indicating the possibility to apply this substance for dental infections as well [27]. The present study aimed to demonstrate the activity of NCT in contaminated dental implant devices. Accordingly, we chose several implant screws commonly used in dentistry. The similar reduction in CFU/mL with all kinds of screws underlines that the bactericidal activity of NCT is independent of the implant material applied. The attachment of plaque bacteria, however, might be influenced a little by the material since we found small but significant differences in the CFU count of the controls. This was seen consistently after the 24 h and 48 h incubation of the screws in bacterial growth solution. Specific studies are required to evaluate if there are differences between implant materials and surfaces relevant for clinical outcomes.

For the production of larger biofilm areas on the test material in our setting, at least 48 h incubation was necessary for streptococci. With *R. aeria*, only small and few spots of biofilm could be achieved and none with *C. ochracea*. Further experimentation would be needed to establish the biofilm conditions for these species and to test older biofilms [29]. In any case, NCT exerted clear killing activity against both planktonic and biofilm bacteria attached to the implant screws. An incubation time of 30 min of 1% NCT was sufficient to eradicate viability to the detection limit. This finding is in accordance with the killing of *Staphylococcus aureus*, *Staphylococcus epidermidis*, and *Pseudomonas aeruginosa* in biofilms by NCT in previous studies [30,31].

It has been shown that biofilms obtained from human dental plaque grown on hydroxyapatite disks increased their resistance to hypochlorite, iodine, and chlorhexidine after growth for 3 weeks before incubation to the antiseptics [29]. This level of resistance remained constant in older biofilms tested up to 8 weeks. It is true that our present study did not investigate long-term plaque biofilms. Previous research, however, demonstrated a constant activity of NCT against 1–14-week-old biofilms of *S. aureus*, *P. aeruginosa*, and *Klebsiella variicola* and up to 7-week-old biofilms of *Candida albicans*, including mixed bacterial and fungal ones using MBEC inoculator plates [32]. This was confirmed recently with 1–8-week-old biofilms of *S. aureus*, coagulase-negative staphylococci, *Klebsiella pneumoniae*, *Acinetobacter baumannii*, and *C. albicans* on titanium disks [33]. Moreover, the mentioned clinical study using 2% and 3% NCT mouth rinses twice daily for 1 min over 4 days resulted in a significant reduction in the vitality of plaque compared to 0.2% chlorhexidine and 0.9% sodium chloride [24]. This indicates that the in vitro findings of NCT against biofilms are basically transferable to the in vivo situation.

The absence of a plaque reduction in this clinical trial may be in agreement with the absence of a morphological destruction of the bacteria in the scanning electron microscopy of the implant surface in the present study after 30 min of incubation in 1% NCT. What we found is a reduction in extracellular matrix. These results are largely in agreement with previous studies. Amorphous bacteria could be observed in *S. aureus* biofilms on titanium alloy disks not before a few hours of incubation time in NCT, while a loss of extracellular matrix became visible already after 1 h [31]. In another study using biofilm on metal disks, a reduction in extracellular matrix became visible after 30 min incubation in 1% NCT as well, while signs for bacterial destruction appeared between 30 min and 1 h depending on the test strains [32]. Therefore, the loss of viability of bacteria occurs earlier than obvious destruction. Discrete changes in bacterial morphology in transmission electron microscopy, however, may be seen in earlier stages of NCT attack [13].

As a mild antiseptic and anti-infective in dentistry, NCT might have several advantages. Its low reactivity and its body-own nature are reasons for its high tolerability shown, for instance, in the oral cavity and paranasal sinuses [19,24,34], and upper and even lower airways [35,36,37] without systemic adverse effects. Another consequence of mild activity could be less influence on the oral microbiome than other antiseptics, which remains to be investigated. It is true that the killing of virulent bacteria by NCT needs longer incubation times than many other antiseptics. It is, however, enhanced by transchlorination reactions in the presence of organic material, which supports sufficient efficacy at the site of application and may render NCT more effective than stronger active chlorine compounds under certain circumstances [9,14]. Another aspect is the anti-inflammatory properties of NCT by the downregulation of proinflammatory cytokines and upregulation of heme oxygenase-1 so it is thought to contribute to the termination of inflammation and has shown therapeutic efficacy in animal inflammation models [38,39,40,41]. All these preconditions and the activity against oral plaque bacteria render NCT an interesting candidate for the future treatment of periodontal disease and peri-implantitis.

## 4. Materials and Methods

### 4.1. Chemicals

*N*-chlorotaurine (NCT) was synthesized at the Institute of Hygiene and Medical Microbiology of the Medical University of Innsbruck as a crystalline sodium salt in pharmaceutical quality (lot 2022-05-22) and stored at minus 20 °C [9]. Use solutions of 1% and 0.1% (55 and 5.5 mM) were freshly prepared in 0.01 M PBS. L-methionine and L-histidine were from Roth GmbH (Karlsruhe, Germany).

### 4.2. Test Bacteria

Clinical isolates of *Rothia aeria*, *Capnocytophaga ochracea*, *Streptococcus oralis*, *Streptococcus sanguinis*, *Streptococcus salivarius*, and *Streptococcus cristatus* deep-frozen for storage were used. Bacteria had been identified by matrix-assisted laser desorption/ionization-time of flight mass spectrometry (MALDI-TOF MS) (Bruker Daltonics, Bremen, Germany) using the direct smear method. A score above 1.7 was considered valid [42]. *C. ochracea* was grown anaerobically on Columbia III blood agar (Becton Dickinson Austria, Vienna, Austria), the other species aerobically on Mueller–Hinton agar (Becton Dickinson). Overnight cultures of about 16 h (40 h for *C. ochracea*) at 37 °C were prepared in tryptic soy broth (Merck, Darmstadt, Germany), leading to counts of colony-forming units (CFUs)/mL of 1.1–3.6 × 10^7^ for *R. aeria*, to 2.0–5.0 × 10^8^ for *C. ochracea*, to 1.2–6.1 × 10^7^ for *S. oralis*, to 1.0–2.0 × 10^8^ for *Streptococcus sanguinis*, to 1.7 × 10^7^–1.7 × 10^8^ for *S. salivarius*, and to 1.3–4.3 × 10^8^ for *S. cristatus*.

### 4.3. Quantitative Killing Assays of Planktonic Bacteria

Solutions of 1% and 0.1% NCT in PBS, each 3.96 mL, at a pH of 7.1 were prepared in glass tubes and kept at room temperature (about 20 °C) or were pre-warmed to 37 °C in a water bath (Lauda Ecoline E100, Lauda Dr. R. Wobser GMBH & CO. KG, D-97922 Lauda-Königshofen, Germany). Controls were prepared in 3.96 mL PBS without NCT. At time point zero, 40 µL of the bacterial cultures were added separately to the NCT or control solutions. After different adjusted incubation times, aliquots of 100 µL were removed and mixed with 900 µL of 1% methionine/1% histidine in distilled water to inactivate NCT [14]. Aliquots of this suspension were spread onto Mueller–Hinton agar plates in duplicate (50 µL each) using an automatic spiral plater (model WASP 2; Don Whitley, Shipley, UK). The detection limit was 100 CFU/mL, taking into account both plates and the previous 10-fold dilution in the inactivating solution. Plates were grown for 24 h at 37 °C before colonies were counted. Plates with no growth or only a low CFU count were grown for up to five days to detect bacteria attenuated but not killed by the treatment.

### 4.4. Quantitative Killing Assays of Planktonic Bacteria on Dental Implant Screws

Bacteria from overnight cultures (10 µL) as described above in ‘test bacteria’ were added to 3 mL of tryptic soy broth in 12-well microtitre plates (VWR^®^ Tissue Culture Plates 734-2324, VWR International GmbH, Vienna, Austria) before the addition of screws. Dental implant screws comprised samples from 4 different companies as listed in Table 2. One screw each was placed subsequently in one well and incubated at 37 °C for 24 h in an incubator without shaking. Then, the screws were removed and placed in tubes containing pre-warmed 1% NCT in PBS and incubated in the water bath at 37 °C for 10 (streptococci) or 20 (*R. aeria*) min. Afterwards, they were removed and placed in 1 mL of 1%methionine/1%histidine solution to immediately inactivate NCT. Controls were prepared in PBS without NCT and run in parallel. The tubes containing the screws in the inactivation solution were vortexed three times for 5 s, sonicated for 1 min in an ultrasound water bath (40 kHz; BactoSonic; Bandelin Electronic, Berlin, Germany), and vortexed again three times to detach the remaining live bacteria from the screws. Test samples were processed undiluted, and controls were 10-fold diluted in 0.9% sodium chloride (100 µL to 900 µL NaCl). Quantitative cultures were performed as detailed above.

### 4.5. Quantitative Killing Assays of Biofilm Bacteria on Dental Implant Screws

Again, 10 µL of overnight bacterial cultures were placed in 3 mL of tryptic soy broth in 12-well plates, followed by the screws. Incubation was performed at 37 °C under continuous shaking (80 rpm, Infors HT Ecotron, Infors AG, Bottmingen, Switzerland) for 48 h. Subsequently, the screws were washed 3 times in 3 mL PBS in 12-well microtitre plates to remove planktonic bacteria before incubation in 1% NCT in PBS for 20 min (controls in plain PBS). This was followed by transfer into inactivation solution, vortexing, ultrasonication, and quantitative cultures as described above.

### 4.6. Electron Microscopy of Biofilm on Dental Implant Screws

A similar procedure of scanning electron microscopy was used as in previous studies on the impact of NCT on biofilms [31,32]. After incubation in NCT or control PBS, the washed implant screws were fixed with 2.5% glutaraldehyde (BioChemika Fluka, Buchs, Switzerland) in 0.1 M phosphate buffer (pH 7.4). After a brief wash in phosphate buffer, the samples were gradually dehydrated with 50%, 70%, 80%, and 99% ethanol. After the last step, the screws were incubated at room temperature for drying out. The dried screws were placed on aluminum pins and fixed with Leit-C (Göcke, Plano GmbH, Wetzlar, Germany). The pins were sputtered with Au 10 nm (Agar Sputter Coater, Agar Scientific Ltd., Stansted, UK) for 1 min and analyzed by scanning electron microscopy (SEM, JSM-6010LV, JEOL GmbH, Freising, Germany).

### 4.7. Statistics

Results are presented as mean values and standard deviation. Student’s unpaired *t*-test for the comparison of two groups and one-way analysis of variance (ANOVA) and Dunnett’s and Tukey’s multiple comparison tests for more than two groups were conducted for the comparison of test samples with controls. *p* values < 0.05 were considered significant.

To obtain an improved comparison of the killing curves of NCT against planktonic bacteria, the Integral Method was used, which transforms the whole killing curve (log10 CFU per ml versus time) into one value of ‘bactericidal activity (BA, log10 CFU per ml per min)’ [15,43]. The method is based on the area below the killing curve, which is calculated by the addition of the areas of trapezoids between the single time points of incubation and transformed into an orthogonal triangle with the same area. Its hypotenuse forms with the abscissa the angle alpha, whose tangent, tg(alpha) = y/2x, represents the sought average BA. The method allows an expanded statistical analysis, particularly between killing curves with small differences [15,43].

## Figures and Tables

**Table 1:** Bactericidal activity (BA) values ^a^ of NCT against planktonic bacteria, calculated with the Integral Method.

Species	1% NCT 37 °C	1% NCT 20 °C	0.1% NCT 37 °C	Controls
*S. sanguinis*	0.4063 ± 0.0395	0.2310 ± 0.0187	0.0891 ± 0.0133	0.0072 ± 0.0100
*S. salivarius*	0.2782 ± 0.0583	0.1998 ± 0.0278	0.0911 ± 0.0116	−0.0120 ± 0.0148
*S. oralis*	0.4009 ± 0.0726	0.3262 ± 0.0465	0.1781 ± 0.0257	−0.0006 ± 0.0152
*S. cristatus*	0.3483 ± 0.0440	0.1669 ± 0.0217	0.1446 ± 0.0152	0.0060 ± 0.0135
*C. ochracea*	0.6239 ± 0.0603	0.4266 ± 0.0346	not done	0.0037 ± 0.0210
*R. aeria*	0.1878 ± 0.0233	0.1262 ± 0.0159	0.0611 ± 0.0079	0.0082 ± 0.0062

^a^ log_10_ reduction in CFU per min. The higher the value, the higher the bactericidal activity. Mean values ± SD calculated from killing curves of Figure 1. Susceptibility: *C. ochracea* > *S. oralis* > *S. sanguinis* > *S. cristatus* > *S. salivarius* > *R. aeria*. *p* < 0.05 for 0.1% NCT versus controls, *p* < 0.01 for 1% NCT versus controls, *p* < 0.05 for 1% NCT versus 0.1% NCT (except for 1% NCT 20 °C for *S. cristatus*), *p* < 0.05 for 1% NCT 37 °C versus 1% NCT 20 °C (except for *S. oralis*).

**Table 2:** Implant screws used for contamination and biofilm tests.

Screw Type	Size	Lot
Easy Dip ^a^	ø 4.75 mm, L 13 mm	Ref. ED47513, lot 2012/0004/1
Easy Dip ^a^	ø 4.75 mm, L 11.5 mm	Ref. ED475115, lot 2012/0033
Easy Dip ^a^	ø 4.75 mm, L 10 mm	Ref. ED47510, lot 2012/0025/2
Carident ^b^	ø 3.6 mm, L 8 mm	Ref: A-03-036080, lot 611600, lot 604194
Carident ^b^	ø 4.2 mm, L 8 mm	Ref: A-03-042080, lot 611602
Carident ^b^	ø 3.6 mm, L 10 mm	Ref: A-03-036100, lot 611601
AnyRidge ^c^	ø 8 mm, L 7 mm	Ref: FALIHX8007C, lot: 170512A1030-01
AnyRidge ^c^	ø 3.5 mm, L 7 mm	Ref: FANIHX3507C, lot: 170427A0440-01
AnyRidge ^c^	ø 6.5 mm, L 10 mm	Ref: FALIHX6510C, lot: 170825A1130-01
AnyRidge ^c^	ø 8 mm, L 13 mm	Ref: FALIHX8013C, lot: 170502A1270-02
Profile1 DeepNeck ^d^	ø 4 mm, L 8 mm	Ref: P1W4008R, lot: 2011/0359/1

^a^ Overmed S.r.l. Buccinasco, Italy. ^b^ Carident AG, Romanshorn, Switzerland. ^c^ MDSS GmbH, Hannover, Germany. ^d^ Profile1, Bcg Technology, Argelato, Italy.

## Data Availability

All data are presented in the article.
